# Intrauterine growth restriction, visceral blood flow velocity and exocrine pancreatic function

**DOI:** 10.1186/1756-0500-1-115

**Published:** 2008-11-17

**Authors:** Richard M Nicholl, Jean M Deenmamode, Harold R Gamsu

**Affiliations:** 1 Neonatal Unit, Kings College Hospital, Denmark Hill, London, SE5 9RS, UK; 2 Dept. Clinical Biochemistry, Kings College Hospital, Denmark Hill, London, SE5 9RS, UK

## Abstract

**Background:**

Animal models and observations in human neonates suggest fetal exocrine pancreas vulnerability to reduced maternofetal blood flow. We investigated the relationship between superior mesenteric artery blood flow velocity (sma bfv) and exocrine pancreatic function, in a cohort of very low birth weight (VLBW) babies.

Group 1: 9 babies < 3rd percentile for birth weight. Antenatally, all had absent or reversed diastolic flow on Doppler ultrasound of the umbilical artery (UA).

Group2: 18 babies > 10th percentile for birth weight.

**Findings:**

All had Doppler ultrasound scan of the superior mesenteric artery (sma), by same operator (RMN), on day 1 of life before commencement of enteral feeding. Stool samples assayed for faecal chymotrypsin and weekly serum samples assayed for amylase and lipase (kinetic colorimetric assay) from days 1 to 14 of life.

Growth restricted babies had significantly lower sma bfv values compared with appropriately grown preterm babies. Faecal chymotrypsin levels were also lower but this difference did not achieve statistical significance. Both groups had serum lipase levels detectable in adult concentrations. Serum amylase was undetectable in either group.

**Conclusion:**

Babies with previous in-utero blood flow redistribution may exhibit altered gut ontogeny with re-setting of mesenteric blood flow velocities and altered exocrine pancreatic function.

## Introduction

Doppler studies of the utero-placental circulation in growth restricted fetuses have shown circulatory redistribution with an increased blood flow velocity in the cerebral circulation and a reduced blood flow velocity in the aorta [1]. This maintains oxygenation and the supply of nutrients to the brain but with the possibility of detriment to other organs, notably those in the abdomen. After birth these babies are at an increased risk for necrotising enterocolitis [2,3].

We have measured biochemical markers of exocrine pancreatic function (faecal chymotrypsin, serum lipase and serum amylase) in a cohort of VLBW babies with both antenatal and post natal Doppler ultrasound evidence of circulatory redistribution.

## Methods

Doppler studies of the superior mesenteric artery were made on 9 VLBW babies below the 3^rd ^percentile for birth weight (growth restricted) and on 18 VLBW babies (control group) with birth weights greater than the 10^th ^percentile. The studies were performed on the first day of life and before enteral feeds had been commenced. In the growth restricted group, growth restriction had been detected antenatally with fetal abdominal circumference on ultrasound examination of less than the 5^th ^centile for gestation. Antenatal Doppler studies in these fetuses had shown absent or reversed end diastolic flow in the fetal aorta, as previously described [2]. Postnatally, blood velocity waveforms were obtained from the superior mesenteric artery using a duplex ultrasound imaging and Doppler system (Hewlett Packard Sonos 100 with 7.5 MHz imaging and 5.0 MHz Doppler transducer). This method, using the time averaged mean of the peak velocity envelope calculated from four consecutive cardiac cycles has been described in detail [4]. Stool samples were collected from days 1–14 and assayed for faecal chymotrypsin (colorometric method of Boehringer Mannheim). Serum samples were analysed weekly for amylase and lipase (kinetic colorimetric assay), also in the first 14 days of life. Ethical approval for the study was given by the Research Ethics Committee of Kings College Hospital and informed written consent was obtained from the parents for each baby studied.

Patient details and results of biochemical assays are shown in the table.

### Statistical interpretation

For gestational age, birth weight and blood flow velocity there was one measurement per baby. For faecal chymotrypsin and lipase there were several measurements per baby over the first 2 weeks of life. These measurements were summarised by calculating the mean value for each baby. Data for faecal chymotrypsin were positively skewed so a log transformation was used on each observation and the mean of the transformed values then calculated. A two sample t test was used to compare the two groups. The blood flow velocity standard deviations differed significantly between the two groups so Satterthwaite's method to perform a t-test was used as this does not assume the same standard deviation in each group.

## Results

**Figure 1:**
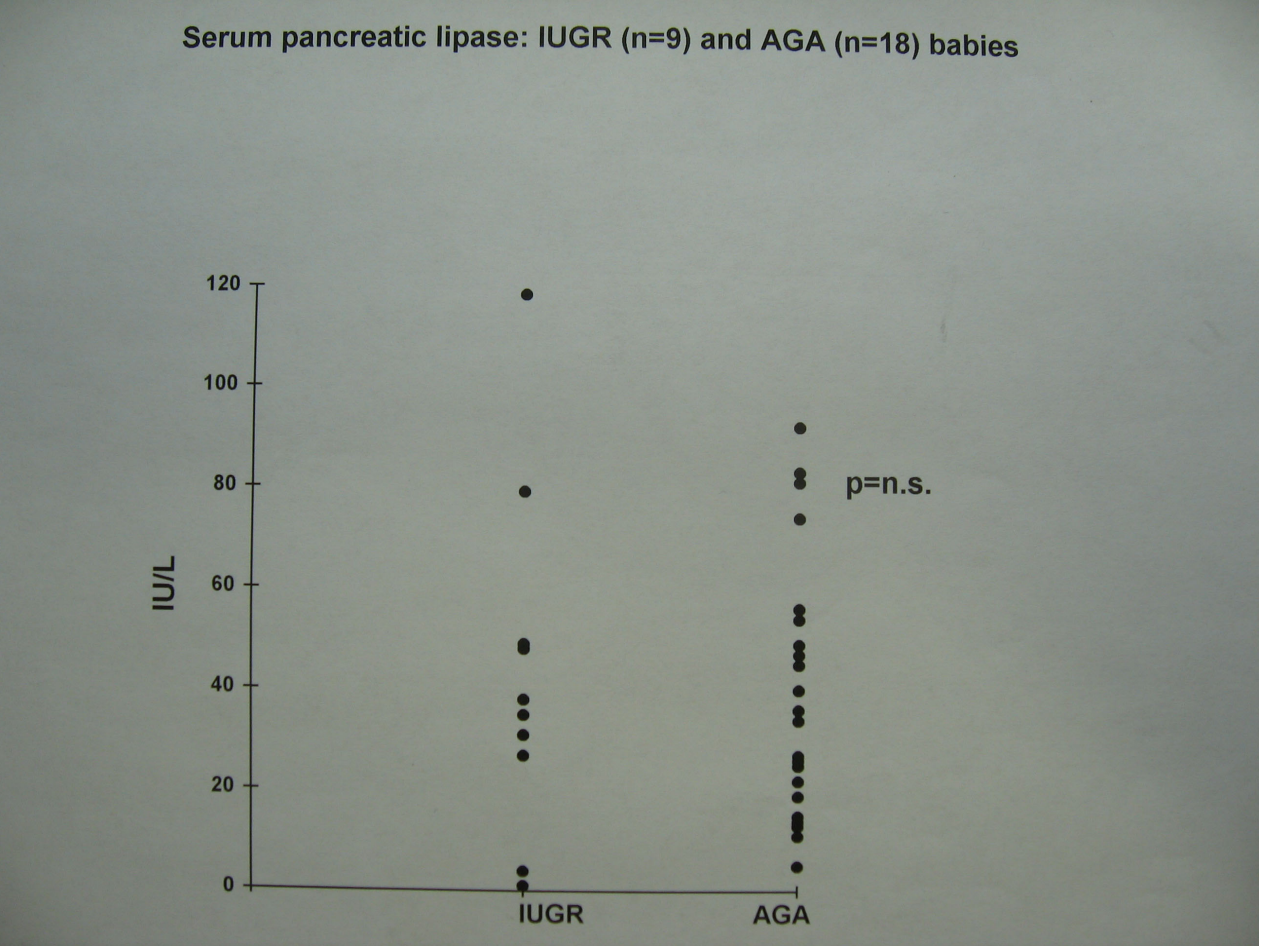
Serum pancreatic lipase: IUGR (n = 9) and AGA (n = 18) babies.

**Figure 2:**
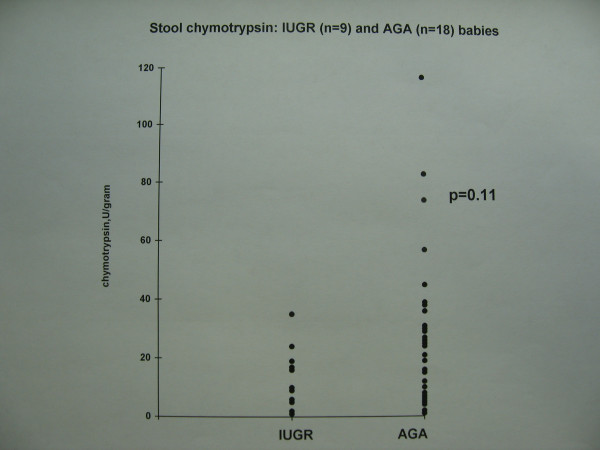
Stool chymotrypsin: IUGR (n = 9) and AGA (n = 18) babies.

**Figure 3:**
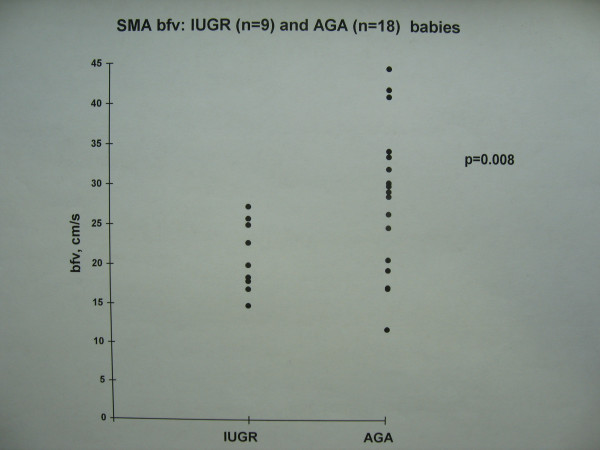
SMA bfv: IUGR (n = 9) and AGA (n = 18) babies.

The growth restricted babies with evidence of antenatal fetal blood flow redistribution had lower sma bfv on the first day of life (Figure 1, Table 1). This difference was statistically significant. Although they also had a trend to lower faecal chymotrypsin levels compared with appropriately grown babies (Figure 2), this difference did not reach statistical significance. Serum lipase was detectable in adult concentrations, but was not related to IUGR (Figure 3) or the presence or type of enteral feed. Serum amylase was undetectable in both groups. There were no episodes of necrotising enterocolitis in either study group. The study was not powered to detect differences in mortality between groups.

**Table 1:** Clinical characteristics, Doppler (superior mesenteric artery blood flow velocity expressed as: sma bfv, cm/sec) and biochemical results for babies studied.

	Group 1 (IUGR) +se	Group2 (AGA) +se	P value
Birth gestation, weeks	31.9 (0.7)	28.2 (0.5)	< 0.001

Birth weight, gm	986 (85)	1159 (69)	0.15

sma bfv, cm/sec	21 (1.5)	28 (2.1)	0.008

Faecal chymotrypsin, U/gm	5.4 (95% CI 2.4–12)	10.8 (95% CI 6.5–17.9)	0.11

Serum lipase, IU/L	36.7 (11)	39.4 (5)	0.81

Serum amylase, IU/L	undetectable	undetectable	

## Discussion

The superior mesenteric artery (sma) supplies the whole of the small intestine (with the exception of the superior part of the duodenum), and also the caecum, ascending colon and most of the transverse colon. Any compromise of this blood supply may have an adverse effect on gut function and perinatal morbidity: post mortem examination has shown that necrotising enterocolitis (NEC) is most likely to occur in the terminal ileum and ascending colon [5].

Growth restricted fetuses have an increased risk of intra-uterine death or asphyxia at birth. Absent or reversed blood flow velocity in the umbilical artery in these fetuses increases the risk of an adverse perinatal outcome such as emergency Caesarean section, neonatal sepsis, intraventricular haemorrhage, respiratory distress, chronic lung disease, acute renal failure, NEC or death (odds ratio 14.2, p < 0.005) [6].

Observations in naturally occurring growth restricted piglets have shown a reduction in gastrointestinal and pancreatic tissue weight [7] and differences in insulin-like growth factors, binding proteins and receptors for growth hormone [8]. An experimental model of reduced maternofetal blood flow in rats [9] led to reduced pancreatic weight and a reduction in fetal amylase and lipase production.

Faecal chymotrypsin is a simple, inexpensive and non-invasive assay that gives a quantitative measure of pancreatic secretory capacity in the preterm newborn. Studies in human neonates have shown a reduction in faecal chymotrypsin (a measure of exocrine pancreatic function [10]) in growth restricted preterm babies compared to appropriately grown preterm babies [11] However, in a case control study of 9 preterm babies who later developed NEC, Wood et al found no difference in neonatal stool chymotrypsin levels between cases and controls matched for gestation and birth weight [12]. Growth restricted babies with lower faecal chymotrypsin levels in early life also had impaired catch up growth at 6 months of life [13]. However, none of the previous studies in the human neonate included the results of antenatal Doppler ultrasound of the fetus, making interpretation of the cause of their growth restriction difficult or at best speculative. We only included growth restricted babies with clear ante natal evidence of compromise to the fetal circulation as shown on Doppler ultrasound.

The lower postnatal sma bfv and lower faecal chymotrypsin levels in these growth restricted babies suggests an adverse effect not just on the perinatal physiology but also on the function of the exocrine pancreas.

This may be a factor contributing to poor postnatal growth, which is often seen in babies with evidence of antenatal circulatory compromise.

## Competing interests

The authors declare that they have no competing interests.

## Authors' contributions

HRG conceived the idea for the study. MJMD carried out all the biochemical analysis. RMN performed all the Doppler scans, wrote the manuscript and is guarantor.
